# The re‐emerging human monkeypox virus: An urgent global health alert

**DOI:** 10.1002/hsr2.928

**Published:** 2022-11-08

**Authors:** Prithiviraj Nagarajan, Leena Rajathy Port Louis, Anusheela Howlader, Kumar Rangarajalu

**Affiliations:** ^1^ Multi‐Disciplinary Centre for Biomedical Research, Aarupadai Veedu Medical College & Hospital Vinayaka Mission's Research Foundation (Deemed to be University) Kirumampakkam, Bahour Puducherry India; ^2^ Department of Pharmacology, Aarupadai Veedu Medical College & Hospital Vinayaka Mission's Research Foundation (Deemed to be University) Kirumampakkam, Bahour Puducherry India; ^3^ Department of Microbiology, Aarupadai Veedu Medical College & Hospital Vinayaka Mission's Research Foundation (Deemed to be University) Kirumampakkam, Bahour Puducherry India; ^4^ Aarupadai Veedu Medical College & Hospital Vinayaka Mission's Research Foundation (Deemed to be University) Kirumampakkam, Bahour Puducherry India

To the Editor,

The coronavirus disease 2019 (COVID‐19) showed that any viral outbreak may cause a pandemic; the periodic outbreaks of novel or reemerging viruses remind us that zoonotic infections will continue to emerge.^1^ In 2022, Monkeypox (MPX) outbreaks were reported in nonendemic places, causing a worldwide wave of public health concern and demands for action from international authorities. On May 2^nd^, 2022, the World Health Organization (WHO) received a report of a case of monkeypox in a patient from United Kingdom who had a travel history to Nigeria. Zoonotic monkeypox disease is endemic in Central and Western Africa.^2^, ^3^, ^4^, ^5^ The monkeypox virus (MPXV), a virus belonging to the genus Orthopoxvirus (OPXV), which also includes variola, the causative agent of smallpox, and resembles smallpox symptoms.^3^, ^4^ The West African and Congo basin clades are the two main groups of MPXV of which the West African clade is the least deadly, with a 1% death rate, and is believed to be responsible for the current pandemic. In the past, the illness was relatively uncommon outside of Africa, with occasional outbreaks mainly in the Democratic Republic of the Congo (DRC) and Nigeria.^2^, ^3^, ^4^ On the current scenario, for a global Public health alert, we further illustrated the historical timeline of outbreaks of the human monkeypox virus until 2022 (Figure 1). Due to interaction with infected pet Prairie dogs imported from Ghana, the first MPX cases in humans were identified in the United States in 2003, resulting in an outbreak of more than 70 cases.^6^, ^7^ A major human MPX epidemic brought on by the West African clade was also reported in Nigeria in October 2017, with approximately 146 clinically suspected and 42 confirmed cases.^8^ As a consequence of MPXV exports from Africa, human MPX cases were later reported in Israel (2018), the United Kingdom (2018, 2019, 2021, and 2022), Singapore (2019), and the United States (2021).^7^, ^9^ Figure 1 shows the historical timeline of human MPX outbreaks; for further references and ideas, we recommend the following resource for viewing updates.^6^ As of August 1^st^, 2022, four deaths have been documented in nonendemic nations (two in Spain, one in Brazil, and one in India), contributing to 10 deaths globally during this COVID‐19 pandemic (four from non endemic countries and six from endemic countries).^10^

**Figure 1:**
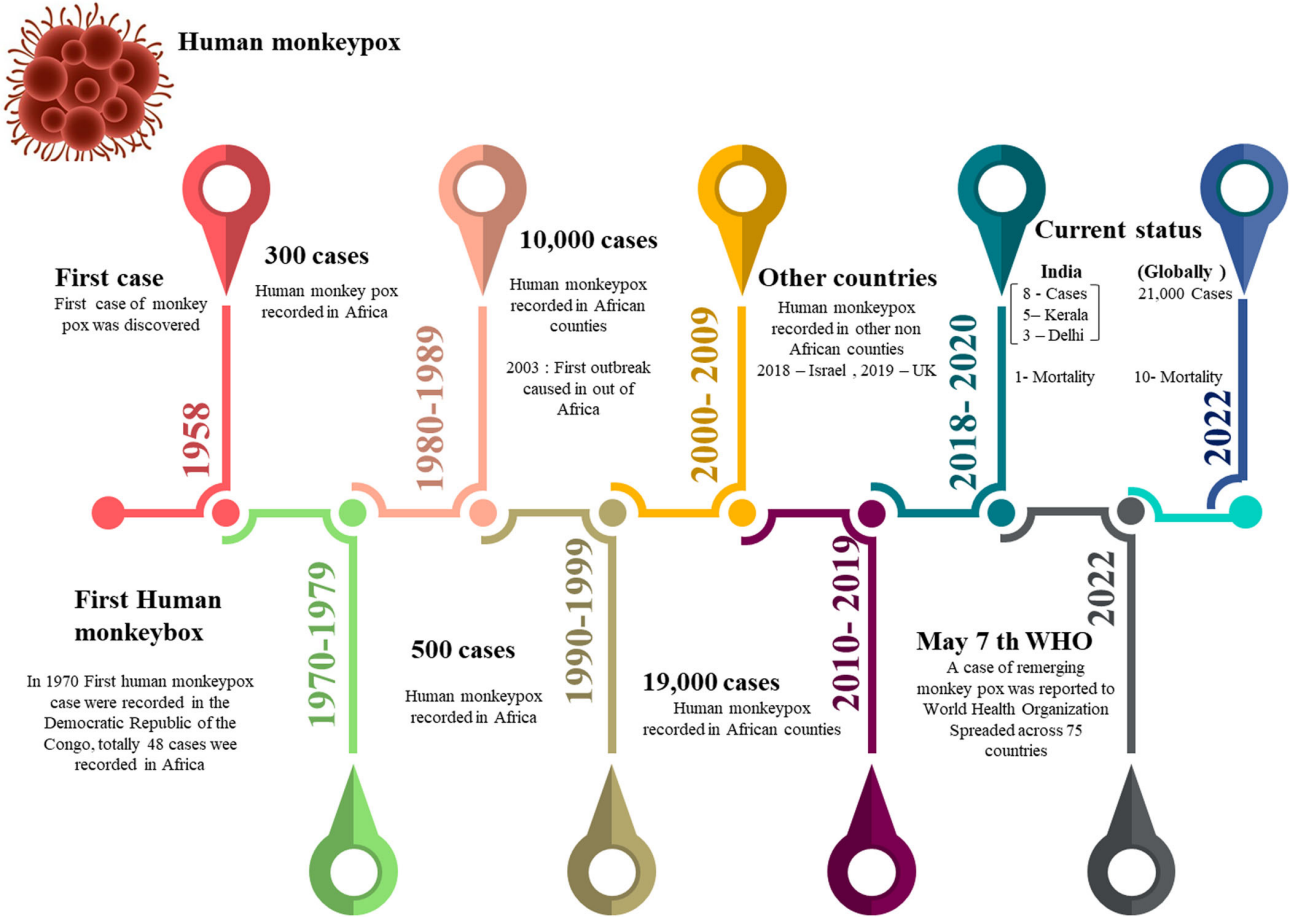
Timeline of reported human monkeypox outbreaks in the World from 1958 till 2022. *Source*: based on data from the Centres for Disease Control and Prevention.

The MPXV transmission to humans is still a mystery. A zoonotic animal‐to‐human transfer may result from direct contact with infected animals (e.g., Bites, Scratches) or indirect contact with contaminated animal fluids or wound material.^11^, ^12^, ^13^ Direct contact with an infected person is the primary mode of transmission by respiratory droplets and exposure to infectious wounds or body fluids.^13^ Human to human transmission occurs via direct skin to skin contact with gaping sores and indirect contact with infected fomites such as bedding or clothes.^14^ Additionally, it is important to consider a vertical transfer from the mother to the fetus.^15^, ^16^ To date, there is no evidence that only human to human transmission in the general population can spread monkeypox infection. Monkeypox transmission in both endemic and nonendemic environments is summarized in Figure 2.

**Figure 2:**
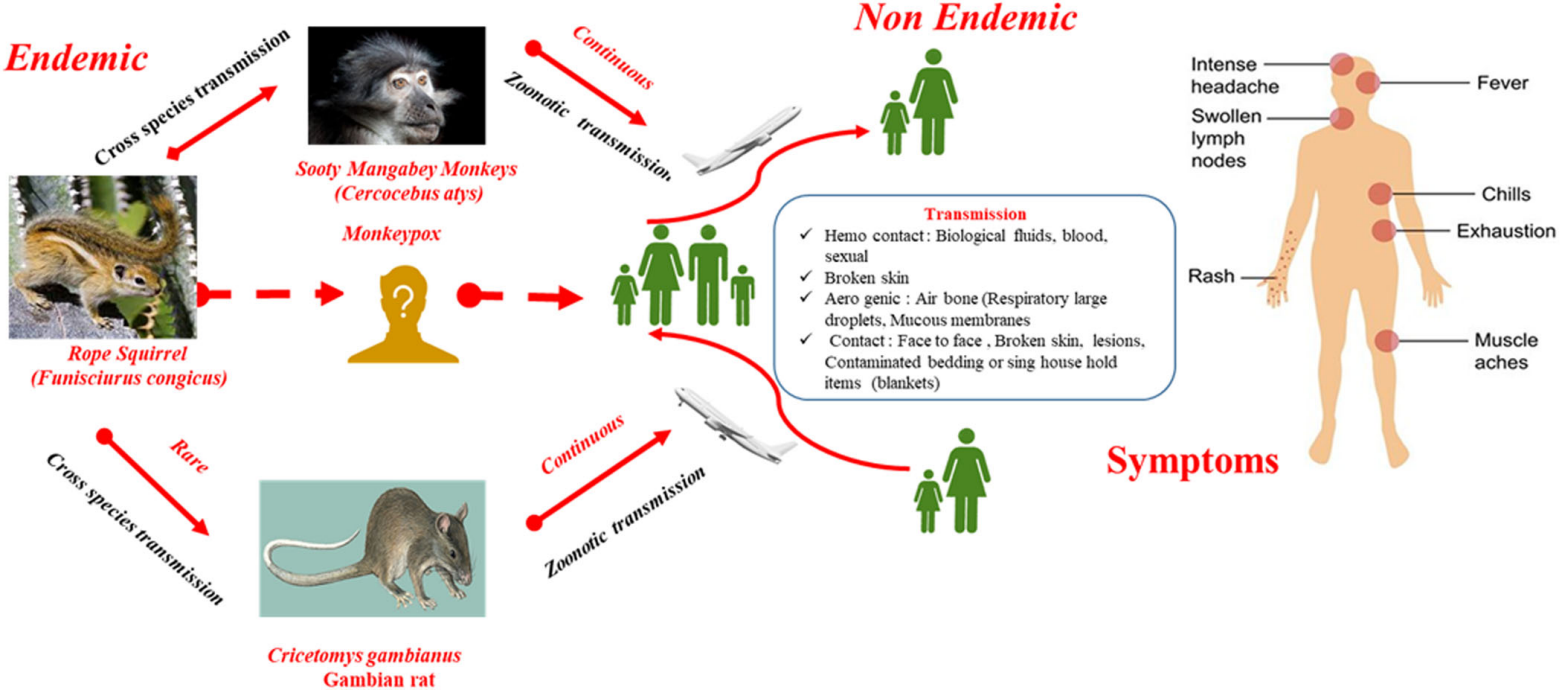
Summarizes the monkeypox transmission in both endemic and nonendemic environments

The typical clinical presentation of monkeypox is characterized by fever, enlarged lymph nodes, and rashes. Prodromal symptoms such as chills, myalgia, fatigue, headache, back pain, and, in rare cases, sore throat and cough may appear.^17^ Many symptoms of monkeypox are similar to those of smallpox.^18^ Itching in the mouth rashes leading to impaired food intake. Secondary bacterial infections of the skin lesions are common in patients.^19^, ^20^, ^21^ The cutaneous signs of monkeypox may be misinterpreted as chickenpox, distinctive rash might be restricted to the vaginal, perigenital, and perianal regions; individuals may also present with/absent or minor prodromal symptoms after a localized rash appears.^22^ Laboratory confirmation can be established using immunological techniques such as ELISA, polymerase chain reaction, electron microscopy, and sequencing.^21^, ^23^

There is no specific therapy for Monkeypox at the moment. The major suggestions for treating MPXV infection are supportive care, symptomatic management, and treatment of subsequent bacterial infections. Since the monkeypox virus is similar to smallpox virus, antiviral drugs developed against smallpox can be used for protection against monkeypox too. Based on smallpox treatment results, antiviral drugs such as Cidofovir, Brincidofovir, and Tecovirimat can be effective against MPXV.^24^, ^25^ Tecovirimat which inhibits viral envelope protein p37 by stopping viral egress from infected cells is approved by the Food and Drug Administration (FDA) for the treatment of smallpox in children and adults. For monkeypox, all antiviral drugs are still investigational drugs that have not been approved by FDA and should be used only in people with severe monkeypox disease or in high risk people with weakened immune system. Under Expanded Access Investigational New Drug (EA‐IND) protocols held by the Centers for Disease Control and Prevention (CDC), Tecovirimat, Cidofovir, and VIGIV are currently accessible from the Strategic National Stockpile for use in treating OPXV infections in an outbreak scenario.^26^ There are now two approved orthopoxvirus vaccines in the United States that can be used to prevent Monkeypox and smallpox. One vaccine (JYNNEOSTM) is based on a live, attenuated vaccinia virus that cannot replicate in the body but may trigger robust immune responses.^27^, ^28^, ^29^ The second vaccine, ACAM2000®, is a replication‐competent live vaccinia virus vaccine, meaning that the vaccine virus may be transmitted from vaccinated to unvaccinated people.^30^ Another vaccine developed to stop viral replication is LC16m8, which protects against severe Monkeypox disease in nonhuman primate animals.^29^ Its effectiveness against human monkeypox disease is yet to be proved.

Preventing infectious disease outbreaks is a major concern for global public health. Reusing Vaccinia Vaccination on a Large Scale should be implemented in affected countries. Furthermore, it is crucial to take preventative actions to minimize zoonotic and human‐to‐human infections.^31^, ^32^ About 75% of today's emerging infectious diseases are zoonotic,^33^ spread by wildlife or exotic pets, such as SARS, Ebola, Salmonellosis, and Monkeypox. Hence, we feel that as most zoonotic diseases have a high chance of spreading through imported exotic pets, strict guidelines to prevent illegal animal traffic and stern animal quarantine procedures for the import of pets from disease‐endemic areas should be implemented worldwide. The CDC says there are several ways to avoid getting infected with MPXV^34^:

1. Avoiding sick animals or anything that has come into contact with a sick animal.
2. Staying away from sick or dead animals in disease‐prone areas.
3. Isolation of the patient.
4. Washing hands after touching contaminated people or animals.
5. Providing medical care while wearing masks and gloves.
6. Public education and awareness can help stop the virus's spread.
7. Infected exotic pets or animals should be quarantined or euthanized during shipping, per CDC guidelines.^35^

The COVID‐19 pandemic has sadly taught us that awareness and preparedness are the two keywords to deal with these dire situations. Scientific community should come up with strategies to develop several therapeutic drugs and vaccines. The country should also increase its immunization units in primary health care centers and hospitals in all areas and improve awareness on public health programs and preventive measures. So far, no promising treatment or prevention strategies have been developed against the human monkeypox virus. From the perspective of the current outbreak, developing an effective vaccine and therapeutic agent against the re‐emerging monkeypox virus is another major challenge for virologists and scientists. Since viruses have evolved in such as way that they are difficult to kill, virologists are considered as key stakeholders in identifying and controlling new emerging viral infections. Therefore, governments have included virologists as key members in pandemic preparedness and response teams worldwide. Although existing smallpox virus replication antiviral agents inhibit orthopoxvirus replication in vitro, developing a new vaccine against all MPX viruses will be the ultimate preventive strategy locally and globally.

## AUTHOR CONTRIBUTIONS

**Dr. Prithiviraj Nagarajan**: Conceptualization; data curation; resources; writing – original draft. **Dr. Leena Rajathy Port Louis**: Data curation; Formal analysis; writing – review & editing. **Dr. Anusheela Howlader**: Formal analysis; investigation; writing – review & editing. **Dr. Kumar Rangarajalu**: Investigation; supervision; validation.

## CONFLICT OF INTEREST

The authors declare no conflict of interest.

## Data Availability

The data sets included in this study are available upon request from the corresponding author.
